# The Evolving Role of TRAFs in Mediating Inflammatory Responses

**DOI:** 10.3389/fimmu.2019.00104

**Published:** 2019-02-04

**Authors:** Bipandeep Dhillon, Fatemah Aleithan, Zahi Abdul-Sater, Ali A. Abdul-Sater

**Affiliations:** ^1^School of Kinesiology and Health Science, Muscle Health Research Centre, York University, Toronto, ON, Canada; ^2^Department of Basic Sciences, Phoenicia University, Mazraat El Daoudiyeh, Lebanon

**Keywords:** inflammation, innate immunity, TRAF, TLRs, NLR, RLR, STING, TNFR

## Abstract

TRAFs [tumor necrosis factor (TNF) receptor associated factors] are a family of signaling molecules that function downstream of multiple receptor signaling pathways and play a pivotal role in the biology of innate, and adaptive immune cells. Following receptor ligation, TRAFs generally function as adapter proteins to mediate the activation of intracellular signaling cascades. With the exception of TRAF1 that lacks a Ring domain, TRAFs have an E3 ubiquitin ligase activity which also contributes to their ability to activate downstream signaling pathways. TRAF-mediated signaling pathways culminate in the activation of several transcription factors, including nuclear factor-κB (NF-κB), mitogen-activated protein kinases (MAPKs; e.g., ERK-1 and ERK-2, JNK, and p38), and interferon-regulatory factors (IRF; e.g., IRF3 and IRF7). The biological role of TRAFs is largely due to their ability to positively or negatively regulate canonical and non-canonical NF-κB signaling. While TRAF-mediated signaling regulates various immune cell functions, this review is focused on the recent advances in our knowledge regarding the molecular mechanisms through which TRAF proteins regulate, positively and negatively, inflammatory signaling pathways, including Toll–IL-1 receptors, RIG-I like receptors, and Nod-like receptors. The review also offers a perspective on the unanswered questions that need to be addressed to fully understand how TRAFs regulate inflammation.

## Introduction

The Tumor-Necrosis Factor (TNF) Receptor Associated Factor (TRAF) family is comprised of cytoplasmic adaptor proteins involved in transducing downstream effects of a variety of receptors (1). TRAF1 and TRAF2 were first discovered through their association with TNF-R2 (2). Since then four other members have been identified, thus, a total of six known members exist (TRAF1 to TRAF6) (3, 4). The TRAF domain can be divided into a N-terminal coiled-coil region (TRAF-N) and a highly conserved C-terminal Beta-sandwich domain (MATH Domain) (4, 5). It is the MATH domain which allows TRAF molecules to form dimers and recruit downstream effectors to receptors (1). With the exception of TRAF1, all other TRAF members, contain a N-terminal RING finger, followed by a variable number of zinc fingers (1, 4, 6). The RING finger motif allows TRAF molecules to act as E3 ubiquitin ligases (5, 6). As adaptor proteins and E3 ubiquitin ligases involved in several immune pathways, TRAFs ultimately lead to the activation of transcription factors, such as nuclear factor-κB (NF-κB), mitogen-activated protein kinases (MAPKs; e.g., ERK-1 and ERK-2, JNK, and p. 38), and interferon-regulatory factors (IRF; e.g., IRF3 and IRF7) (5, 6). In addition, TRAF proteins play important roles in embryonic development, tissue homeostasis, stress response, and bone metabolism (3, 6). Since being discovered in TNF receptor signaling, TRAFs' role has expanded to include involvement in many other inflammatory signaling pathways such as toll-like receptors (TLRs), nucleotide binding-oligomerization domain (NOD)-like receptors (NLRs), retinoic acid-inducible gene I (RIG-I)-like receptors (RLRs), and cytokine receptors (4, 6). Aberrant and prolonged activation of inflammation following the activation of these receptors has been associated with debilitating diseases including cancer, atherosclerosis, type II diabetes, and autoimmune diseases (7). Therefore, a number of mechanisms have evolved to negatively regulate these pathways (8). This review is focused on recent studies that identified new roles for TRAF proteins in activating and inhibiting TLR, RLR, and NLR signaling, and emphasizes newly discovered mechanisms of regulating these pathways by targeting TRAF expression and function.

## The role of TRAFs in Toll-Like Receptor signaling

**Figure 1:**
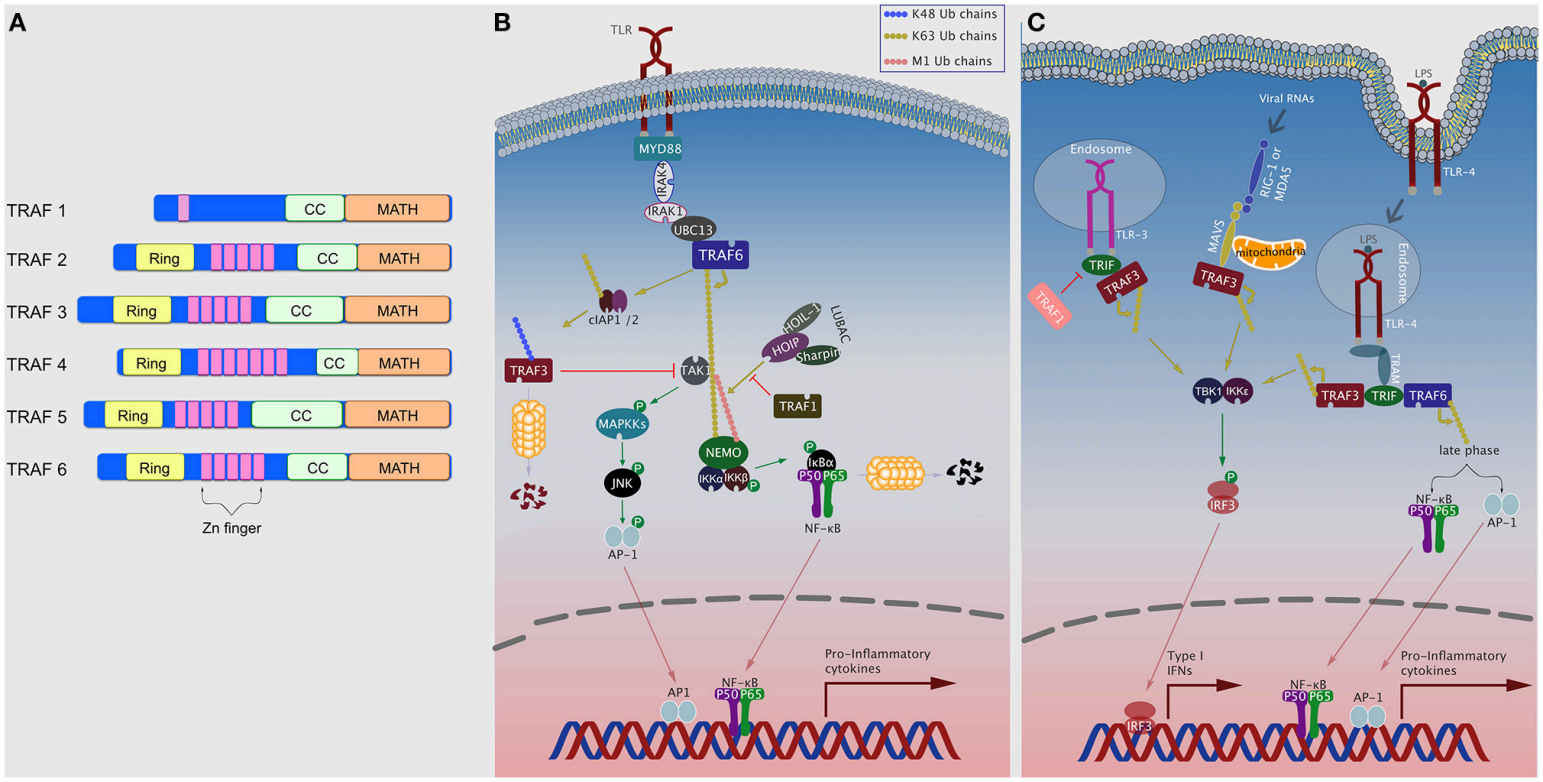
TRAFs in TLR signaling pathways. **(A)** Domain organization of TRAF proteins. Domains shown include Ring, Zinc (Zn) finger, coiled-coil (CC; TRAF-N), and MATH (TRAF-C) **(B)** Plasma membrane TLRs, upon ligand binding, recruit various intracellular signaling elements including TRAF6 to activate NF-κB and MAPK pathways. TRAF1, TRAF3, TRAF4, and TRAF5 can negatively regulate this pathway by different means. **(C)** Following ligand binding, TLR4 translocates to the endosomal compartment and recruits TRIF and TRAF3 to induce the TBK1/IKKε/IRF3 axis, or TRAF6 to induce NF-κB and AP-1 via late phase (slower) kinetics. TLR3 and RLRs can also induce the TBK1/IKKε/IRF3 axis by recruiting TRAF3.

Toll-like receptors (TLRs) are a family of transmembrane receptors lining both cellular and endosomal membranes that sense various pathogen-associated-molecular patterns (PAMPs), and danger-associated molecular patterns (DAMPS) (6, 9–11). There are 10 known TLRs in humans that either exist as homo or heterodimers (11). TLRs are characterized by an extracellular ectodomain comprised of leucine-rich repeats (LRRs), which senses the corresponding PAMP or DAMP, a transmembrane domain, and an intracellular Toll/IL-1 receptor (TIR) domain, which induces the downstream response (9, 12). Upon stimulation, TLRs oligomerize, and recruit MyD88, with the exception of TLR3, which recruits TRIF through TIR domain interaction (12). TLR4 can uniquely induce both MyD88-dependent signaling when it's on the plasma membrane and TRIF-mediated signaling when translocated to the endosomal compartment. Subsequently, a signaling cascade is initiated which results in the activation of transcription factors like NF-κB, MAPKs, and IRFs. This ultimately leads to the production of chemokines, cytokines, and other inflammatory mediators, which initiate the innate immune response and prime the adaptive immune response (Figure 1) (6, 9, 13).

MyD88-dependent signaling is initiated with the recruitment of the IL-1 receptor-associated kinase (IRAK) 4 which, in turn, recruits and activates, through phosphorylation, IRAK1 and IRAK2 (14). IRAK1/2 recruit TRAF6, which functions as an E3 ubiquitin ligase following its oligomerization via the CC domains (15). This also allows TRAF6 to associate with the E2 ubiquitin complex Uev1A:Ubc13, which together then catalyze the K63-linked polyubiquitination to TRAF6 and other substrates, including TAK1, TAB1, TAB2, and NEMO (IKKγ) (16). This activates TAK1 which co-localizes with the IKK complex and activate IKKβ via phosphorylation (9, 17). Importantly, optimal activation of the IKK complex requires the linear ubiquitination of NEMO (M1-linked) (18–20). This is mediated by a ubiquitin ligase complex termed the Linear UBiquitin chain Assembly Complex (LUBAC) and consists of heme-oxidized IRP2 ubiquitin ligase-1 (HOIL-1), HOIL-1–interacting protein (HOIP), and the Shank-associated RH domain interactor (SHARPIN) (21). Activation of the IKK complex leads to phosphorylation and subsequent degradation of the inhibitor of κB, IκBα, which eventually leads to NF-κB activation (12, 13). TAK1 also induces activation of MAPKs, such as ERK1/2, P38, and JNK, through phosphorylation leading to activation of transcription factors like AP-1 (Figure 1A) (12).

In TRIF-mediated signaling, TRIF recruits TRAF3, which catalyzes its own K63-linked polyubiquitination. This leads to the activation of the TBK1 and the non-canonical IKK, IKKε, which in turn phosphorylates IRF3 resulting in its nuclear translocation and the subsequent induction of type 1 IFNs (IFN-Is) (22, 23). With slower kinetics (i.e., late phase), TRIF can also form a complex with TRAF6 and RIP1, to induce the TAK1/IKK axis and the subsequent activation of NF-κB (Figure 1B) (11). IFN-Is can also be induced following TLR7 and TLR9 stimulation through the MyD88-dependent pathway. MyD88 forms a complex with TRAF3 which then recruits and activates an IRAK-IKKα complex, which in turn phosphorylates IRF7 resulting in its translocation into the nucleus to induce interferon production (Figure 1C) (9).

### TRAFs Negatively Regulate TLR Signaling

In addition to activating TLRs, TRAFs can also function as negative regulators of TLR signaling. TRAF3 negatively regulates TLR-mediated MAPK activity, possibly by preventing the release of the TRAF6:TAB1/2:TAK1 complex, but the negative regulation is inhibited by cIAP1/2 which catalyze K48-polyubiquitinated degradation of TRAF3 (Figure 1A) (11, 23, 24). Under specific conditions, TRAF2 can dampen TLR mediated cytokine production by causing proteasome-dependent degradation of c-Rel, a member of the NF-κB family, in a mechanism that also requires TRAF3 and cIAP1/2 (25). TRAF5 has also been shown to inhibit TLR-stimulated cytokine production by preventing the interaction between TRAF6 and TAB2 (26). TRAF4 associates with p47phox, a component of cytosolic NADPH oxidase, to negatively regulate TLR signaling by interacting with TRAF6 and TRIF and disrupting their functions (27). Recently, TRAF1 has been shown to attenuate TLR-induced NF-κB signaling by interfering with LUBAC-mediated linear ubiquitination of NEMO (Figure 1A) (28). Interestingly, downstream of TLR3 signaling, TRAF1 inhibits TRIF mediated activation of NF-κB, ISRE, and the IFN-β promoter independent of IRF-3 (Figure 1B) (29).

### Negative Regulation of TLR Signaling by Targeting TRAFs

TLR signaling can also be regulated by targeting the function, expression, or catalytic activity of certain TRAF proteins. Several deubiquitinases (DUB) have been shown to negatively regulate TLR signaling by removing ubiquitin chains from TRAFs or their targets. For instance, A20 is a key regulator of TLR signaling, whereby it targets several aspects of the signaling cascade. It can accomplish this, in part, by directly deubiquitinating TRAF6 (30). Monocyte chemotactic protein-induced protein 1 (MCPIP1) is another DUB that negatively regulates JNK and NF-κB signaling by deubiquitinating TRAF2, TRAF3, and TRAF6 (31). Recently, peroxiredoxin-1 (PRDX1) has been shown to directly interact with TRAF6 ring finger motif and inhibit its ubiquitin-ligase activity, which diminishes NF-κB activation downstream of TLR4 stimulation [(32), p. 1]. Several members of the NLR family, discussed below, have been shown to regulate TLR signaling by targeting TRAF proteins. NLRC3 can attenuate TLR-mediated NF-κB activation by reducing K63-linked polyubiquitination of TRAF6 (33). NLRX1 can interact with TRAF6 to reduce canonical NF-κB activation through the TLR4 mediated pathway [(34), p. 1]. Under normal conditions, NLRX1 associates with TRAF6, but upon TLR4 stimulation, NLRX1 dissociates from TRAF6 and binds to NEMO preventing TRAF6 recruitment of the IKK complex, and subsequent NF-κB activation (35). It's important to note, however, that some of those findings have been controversial in the field as other studies were not able to reach a similar conclusion [(36, 37); (38), p. 1]. NLRP11 inhibits TLR signaling by recruiting RNF19A, an ubiquitin ligase, to catalyze K48-linked polyubiquitination and the subsequent degradation of TRAF6 [(39), p. 11]. NLRP12 reduces non-canonical NF-κB stimulation by interacting with TRAF3 and NIK causing NIK is degraded preventing/reducing cleavage of p100 to p52 (40).

## The role of TRAFs in NOD-Like Receptor signaling

NOD-like receptors (NLRs) are a family of cytosolic receptors that sample intracellular PAMPs and DAMPs (41–44). These receptors participate in a plethora of cellular processes including: inflammasome assembly, pyroptosis, activation of NF-κB and MAPK pathways, autophagy, IFN signaling, antigen processing and presentation, and ROS production (6, 43, 45). These receptors are characterized by a central nucleotide binding (NOD, also known as NACHT) domain, which allows oligomerization, followed by a C-terminal leucine-rich repeat (LRR) domain, which detects PAMPs and DAMPs and a variable N-terminal domains, which helps induce the downstream response (41–44, 46).

**Figure 2:**
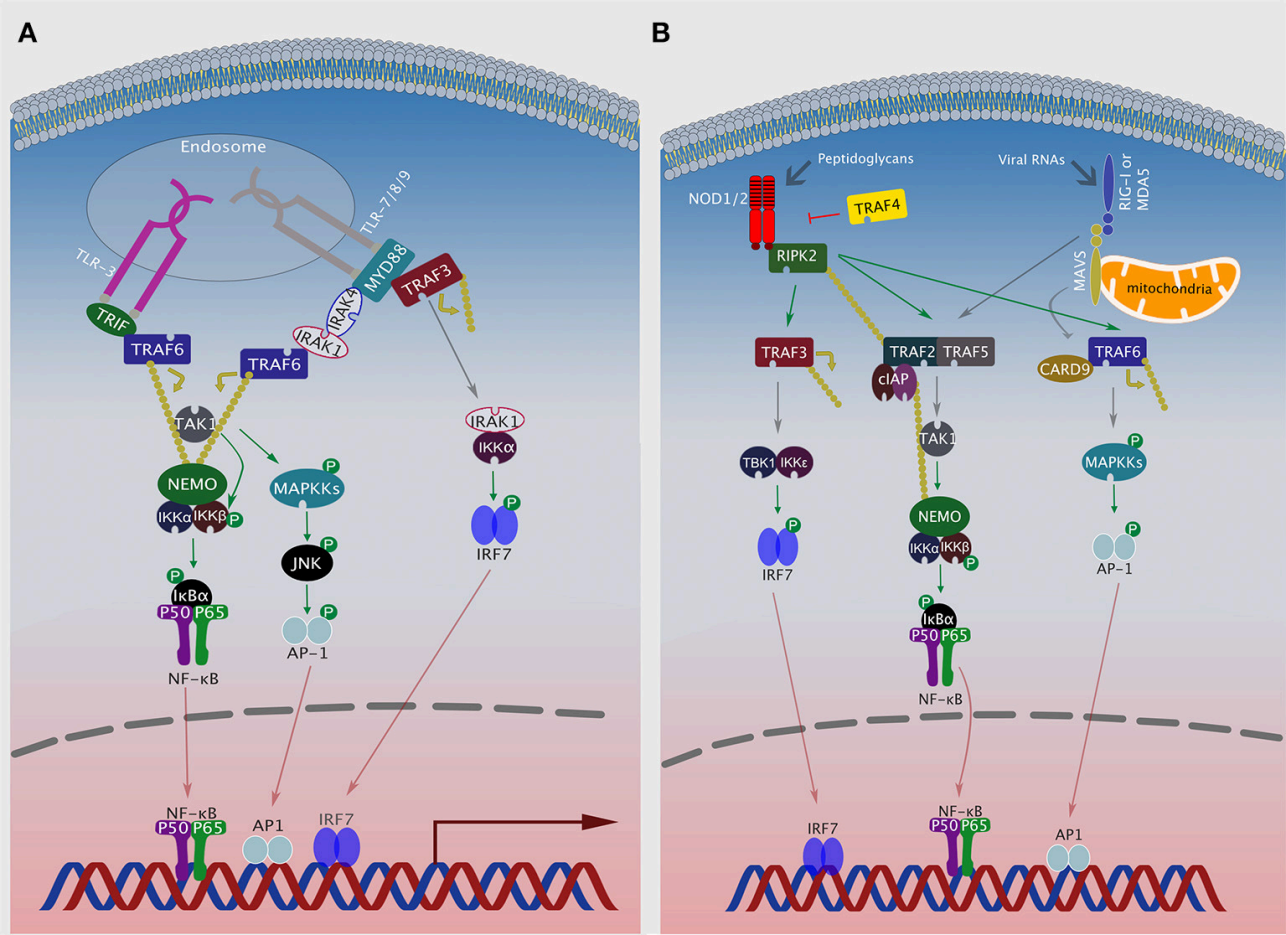
TRAFs in NLR and RLR signaling pathways. **(A)** After ligand recognition, endosomal TLRs recruit TRAF6, either via TRIF (TLR3) or via MyD88/IRAK1/IRAK4 (TLR7, 8 or 9) to activate NF-κB. Additionally, TLR 7, 8, or 9 can recruit MyD88, TRAF3, IRAK1, and IKKα to activate IRF7. **(B)** Ligand activated NOD1 or NOD2 associate with RIPK2, which can then recruit either TRAF3 to activate the TBK1/IKKε/IRF7 axis, TRAF2, and TRAF5 to activate NF-κB or TRAF6 and CARD9 to activate MAPK signaling. Viral RNAs activate RIG-I or MDA5, which then associate with the mitochondrial protein MAVS and activate NF-κB or MAPK signaling by recruiting TRAF2/5 or TRAF6/CARD9, respectively.

NOD1 and NOD2 are the most studied members of the NLR family [reviewed in Motta et al. (47)]. Upon activation, these receptors oligomerize through their NACHT domains and form a complex with RIPK2, through homotypic CARD-CARD interactions (44). In this complex, RIP2 is associated with multiple E3 ligases, including cIAP1/2, xIAP, TRAF2, and TRAF5, but only cIAP1/2 catalyzes its K63-linked polyubiquitination (44, 48–50). TRAF2 and TRAF5 serve as adaptor molecules to facilitate interaction of cIAP1/2 with RIP2 in this complex (48). RIPK2 then induces K63-linked polyubiquitination of TAK1 and NEMO, which recruits the IKK complex to the platform leading to IKKβ phosphorylation by TAK1 (43, 44). TRAF6 and CARD9 serve as adaptors which allow NOD1 and NOD2 to induce MAPKs and subsequently activate AP-1 transcription factor (51, 52). In addition to NF-κB and MAPK activation, NOD1 and NOD2, induce IFN-I production by forming a complex with RIPK2. This results in the recruitment of TRAF3 leading to the activation of TBK1/IKKε/IRF7 axis (Figure 2) (42, 53).

A few NLR family members (e.g., NLRP1, NLRP3, NLRP6, NLRC4, and NLRC5) are capable of activating inflammasomes (41, 54). Inflammasomes are multimeric protein complexes that play a key role in regulating the secretion of potent cytokines like IL-1β and IL-18. Most inflammasomes are composed of an NLR protein, the zymogen pro-caspase-1, and the adapter protein, apoptosis-associated speck-like protein containing a CARD (ASC) (55, 56). Intriguingly, TRAF3 has been recently shown to play a role in NLRP3 inflammasome activation, as it catalyzes K63-linked polyubiquitination of ASC in order to induce ASC speck formation and inflammasome activation (57). In addition, TRAF2, along with cIAP1/2, mediates K63-linked polyubiquitination of caspase-1 for optimum activity (58). However, TRAF2^−/−^ bone marrow-derived macrophages (BMDMs) show normal inflammasome activation, suggesting that TRAF2 may not even be involved in this pathway (59). Following TLR signaling, TRAF6 promotes the non-transcriptional priming of NLRP3 by inducing its oligomerization and association with ASC (60).

### TRAFs Negatively Regulate NLR Signaling

TRAF4 has been shown to act as a negative regulator in NOD2-mediated NF-κB signaling by direct interaction with NOD2. This interaction then allows IKKα to phosphorylate TRAF4, which results in its dissociation from NOD2 and inhibition of NOD2 signaling (61). NLRC3 has been shown to attenuate NLRP3 inflammasome by with ASC for pro-caspase-1 binding (62).

## The role of TRAFs in RIG-I-Like Receptor signaling

RIG-I-Like receptors (RLRs) are a family of DEAD box helicases that play a crucial role in the innate immune response to viral infections by detecting the presence of viral RNA in the cytosol. RIG-I and MDA5 are the two prototypical members of the RLR family (63). Upon sensing viral RNAs, RLRs dimerize and interact with mitochondrial antiviral signaling adaptor (MAVS, a.k.a. IPS-1, or VISA), with the subsequent formation of a complex that includes among others TRAF2, TRAF3, TRAF5, and TRAF6 [(64); (65), p. 3; (66)]. TRAF proteins then recruit various downstream signaling proteins that culminate in the activation of several transcription factors, including IRF3, NF-κB, and MAPKs.

RIG-I/MDA5 employ TRAF3 to induce IRF3-mediated IFN-I production. Mechanistically, TRAF3 is recruited by MAVS, where it catalyzes its own K63 polyubiquitination followed by recruitment and subsequent activation of TBK1 and IKKε (Figure 1B) (67). TRAF2 and TRAF5 play a crucial role in mediating NF-κB activation after RLRs bind their viral PAMPs, albeit the mechanism remains poorly understood (64, 68). TRAF6 can also be recruited via MAVS, where it activates the TBK1/IKKε/IRF7 as well as the MAPKs/AP-1 signaling axes (Figure 2) (66, 69). Intriguingly, the RIG-I-MAVS-TRAF6 signaling axis leads to IKKβ-dependent phosphorylation of NF-κB (70). Furthermore, RIG-I-MAVS-TRAF6 signaling induces K63-ubiquitination of Beclin-1, a critical step for inducing autophagy (71). Finally, TRAF6 interacts with Ubiquitin-specific protease 4 (USP4) to induce NF-κB activation following RLR-simulation (72). This is achieved via targeting of TRAF6 for K48-linked deubiquitination.

### Regulation of RLR Signaling by Modulating TRAF Function or Its Interactions

During bacterial infections, the E3 ligase HCTD3 adds K63-linked ubiquitin chains to TRAF3, which enhances the activation of the TBK1/IKKε complex and subsequent production of IFN-Is (73). Conversely, several deubiquitinases, including OTUB1, OTUB2, DUBA, and HSCARG, have been shown to downregulate RLR-mediated IFN-I production by removing K63-linked polyubiquitin chains from TRAF3 or TRAF6 [(74, 75), p. 1; (76)]. MCPIP1, which is known to inhibit JNK and NF-κB signaling by deubiquitinating several TRAFs [see above; (31)], has been recently shown to negatively regulate IFNβ production. Overexpression studies showed that MCPIP1 disrupts the interaction between TRAF3, TBK1, and IKKε, as shown by co-immunoprecipitation, and thereby inhibiting the phosphorylation and translocation of IRF3 into the nucleus (77). There was no evidence to show that this process requires the deubiquitinase activity of MCPIP1. Another deubiquitinase, OTU deubiquitinase 1 (OTUD1), has also been demonstrated to attenuate IFN-I production following RIG-I activation by viral RNAs (78). Mechanistically, OTUD1 deubiquitinates and stabilizes the ubiquitin ligase, Smurf1, which then targets the MAVS/TRAF3/TRAF6 signalosome by mediating K48-linked polyubiquitination and the subsequent degradation of MAVS, TRAF3, and TRAF6 (78). Parkin is another ubiquitin ligase that targets RLR signaling by promoting K48-linked polyubiquitination of TRAF3 and reducing its stability (79). An interesting study demonstrated that linear ubiquitination of NEMO promotes its interaction with TRAF3, which in turn, disrupts the recruitment of TRAF3 to the RIG-I/MAVS complex leading to diminished IFN-I expression (80).

## The role of TRAFs in STING signaling

In addition to cytosolic sensors of RNA, DNA sensors in the cytosol are equally crucial in detecting and mounting an inflammatory response against viral and bacterial pathogens. Stimulator of Interferon Genes (STING) is activated directly by second messengers like bacterial cyclic dinucleotides (e.g., c-diAMP and c-diGMP) (81–83) or by cellular cyclic GMP-AMP (cGAMP), which is produced by cyclic guanosine monophosphate (GMP)-adenosine monophosphate (AMP) synthase (cGAS) upon sensing cytosolic DNA (84). Activation of STING leads to an effective inflammatory response which include the activation of the TBK-1/IRF3 and NF-κB axes. TRAF3 and TRAF6 have been shown to enhance STING-mediated NF-κB and IFN-β promoter activity, albeit in an overexpression system in 293 cells (85). Both TRAF3 and TRAF6 appear to interact with STING (85, 86). Recently, an alternative STING pathway has been revealed in keratinocytes in response to DNA damage. This alternative STING signaling complex includes the tumor suppressor p53 and TRAF6, whereby TRAF6 catalyzes K63-polyubiquitination of STING and activates NF-κB (87). An elegant study by Genhong Cheng's group recently showed that the alternative NF-κB inducing kinase (NIK) can associate with STING and enhance its activation via an alternative NF-κB pathway-independent mechanism (88). Interestingly, they showed that TRAF3, unlike its positive role in RNA-induced IFN-I production, plays an opposite role in the DNA pathway and inhibit IFN-I production by suppressing NIK expression (88).

## Perspectives and Future Directions

There continues to be a great interest in understanding how TRAFs regulate innate immune signaling. Specifically, novel mechanisms have been recently identified to regulate TLR, NLR, and RLR pathways by modulating the ubiquitination status of TRAFs, their stability or their function. However, as most of these regulators seem to be non-redundant, investigating additional novel regulators, and their mechanism of action remains an active area of investigation.

Each individual TRAF protein plays several, sometimes contradictory, roles that are pathway and/or cell specific. For example, a particular TRAF protein might induce lymphocyte survival and maturation while inhibiting a certain inflammatory pathway. Furthermore, most TRAFs function as E3 ligases as well as adapter proteins. Therefore, TRAFs are poor candidates for novel therapies since targeting a TRAF protein could lead to unintended consequences. For these reasons, future studies should focus on assessing the various roles of each TRAF protein in isolation from its other functions. This is especially important when designing therapies for complex inflammatory and autoimmune diseases by targeting TRAFs.

## Author Contributions

All authors listed have made a substantial, direct and intellectual contribution to the work, and approved it for publication. BD, FA and AA-S wrote the text. A-AS edited the manuscript and conceptualized the figures. ZA-S created the figures.

### Conflict of Interest Statement

The authors declare that the research was conducted in the absence of any commercial or financial relationships that could be construed as a potential conflict of interest.
